# Enhanced wet grip with North American river otter paws

**DOI:** 10.1111/nyas.15263

**Published:** 2024-12-12

**Authors:** Alexis C. Noel, Jason Lieb, Benjamin Seleb, Mary Thatcher, Soohwan Kim, Aqua T. Asberry, Jason H. Nadler, David L. Hu

**Affiliations:** ^1^ Aerospace, Transportation and Advanced Systems Laboratory, Aerospace & Acoustics Technologies Division Georgia Tech Research Institute Smyrna Georgia USA; ^2^ Woodruff School of Mechanical Engineering Georgia Institute of Technology Atlanta Georgia USA; ^3^ The Petit Institute for Bioengineering and Biosciences Atlanta Georgia USA; ^4^ Advanced Concepts Laboratory, Structures and Applied Materials Branch Georgia Tech Research Institute Atlanta Georgia USA; ^5^ School of Biological Sciences Georgia Institute of Technology Atlanta Georgia USA

**Keywords:** adhesion, grip, *Lontra canadensis*, otter, paw, plantar pad, soft

## Abstract

The semi‐aquatic North American river otter (*Lontra canadensis*) has the unique challenge of navigating slippery algae‐coated rocks. Unlike other river otter species, each rear paw of the North American river otter has a series of soft, circular, and keratinized plantar pads similar to the felt pads on the boots of fly fishermen. Surrounding these soft pads is a textured epidermal layer. In this combined experimental and numerical study, we investigate the influence of the plantar pads and surrounding skin on the otter's grip. We filmed an otter walking and performed materials testing and histology on preserved otter paws. We present experiments and numerical modeling of how the otter paw may help evacuate water when contacting the river bed. We hope this study will draw interest into natural amphibious grip mechanisms for use in sports and the military.

## Graphical abstract

The semi‐aquatic North American river otter (*Lontra canadensis*) has four keratinized, soft plantar pads on each rear paw to enhance grip on wet, slippery substrates. In this combined experimental and numerical study, we investigate how the otter's paw pads and textured skin help evacuate water during locomotion. This work can inspire innovation in anti‐slip solutions for wet environments across robotics, sports, and medicine.

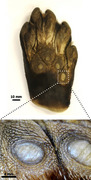

## INTRODUCTION

Animals have evolved diverse multimodal mechanisms to grip a variety of substrate types, either for locomotion or prey capture. For example, geckos use claws and microscopic hair‐like setae to attach to dry surfaces,^1^ remora use suction and mucus to grip shark skin,^2^ and frogs use soft tissue, mucus, and texture to grip both hydrophobic prey^3^ and wet surfaces.^4^ Semi‐aquatic mammals commonly grip slippery substrates. They may use claws as cleats and soft, large paws to help distribute weight.^5^ For instance, polar bears traverse slippery snow and ice‐covered surfaces using rough papillae on their paws.^6^ In this study, we investigate the North American river otter (*Lontra canadensis*), a carnivorous mustelid in the Lutrinae subfamily. From here on, we refer to this species as “the river otter.” It is one of 13 otter species, but unlike its sea‐dwelling cousins, the river otter regularly navigates slippery, biofouled rocks. This semi‐aquatic mammal is found on the waterways and coasts of North America, and can weigh between 5 and 13.6 kg.^7^

**FIGURE 1:**
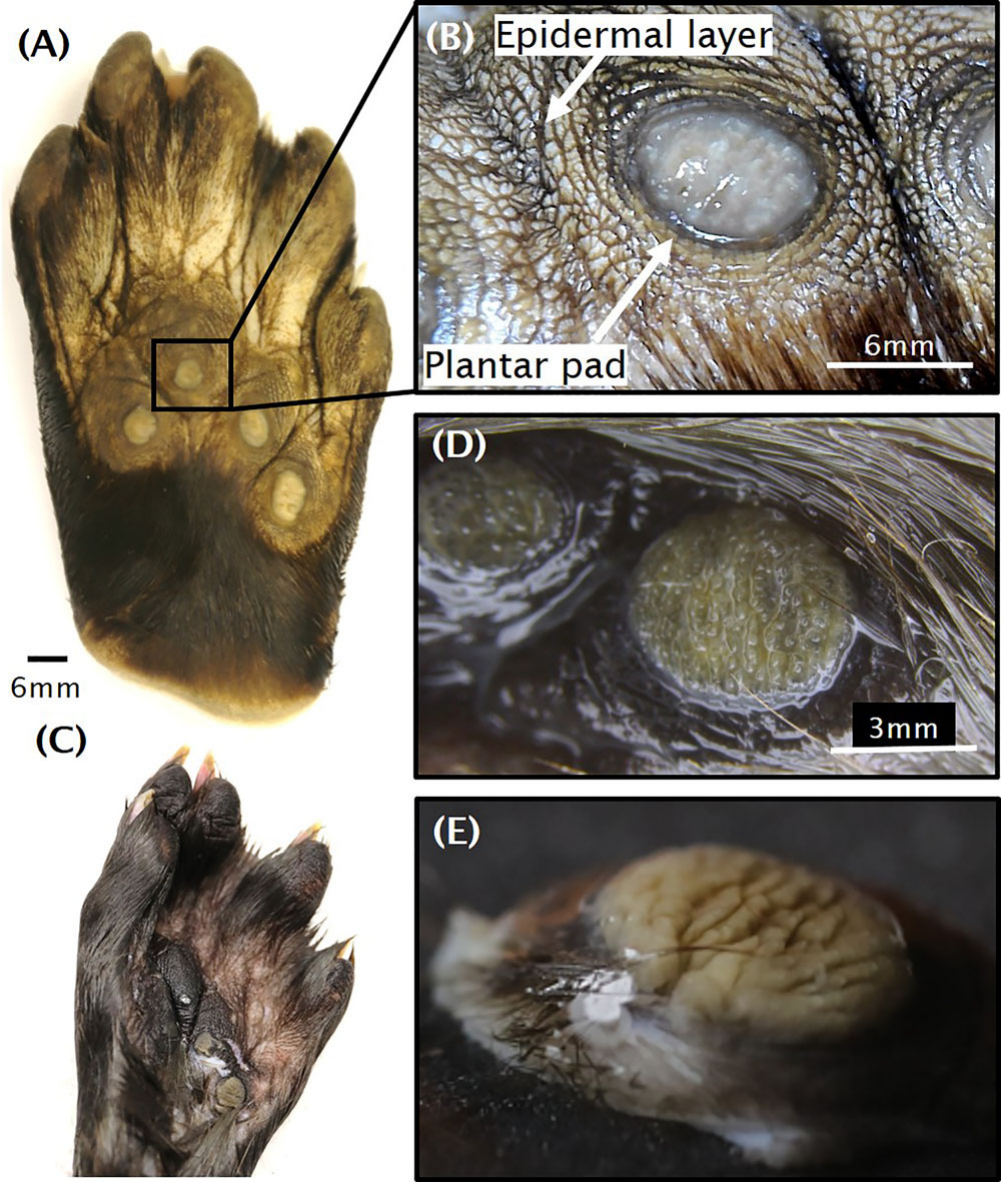
Rear paw pad of a North American river otter. (A) Taxidermied paw of an adult otter. (B) Magnification of the paw pad, showing the white plantar pad surrounded by a textured epidermal layer. (C) Juvenile otter paw from a frozen specimen. (D) Plantar pad of a juvenile otter paw. (E) Oblique view of hydrated pad from panel D, showing cracks.

In general, otters use claws and textured paws to grip onto wet surfaces. On its rear paws, the river otter has plantar pads, a series of four white, oval, felt‐like pads, each around the size of a pencil eraser,^8^ as shown in Figure 1A,B. We hypothesize that these pads are important for animals that spend time in shallow water or on the ground: conversely, sea otters, which live mostly in the open ocean, do not have plantar pads. Plantar pads have been found in other North American mustelids, such as the American marten (*Martes americana*), fisher (*M. pennanti*), and wolverine (*Gulo gulo*). The function of mustelid plantar pads is still debated, but Buskirk postulated that they serve as gland‐based chemical transmitters.^9^ Contact with the ground enables the chemicals to be deposited, similar to the function of musk glands in deer.

This study is motivated by Buskirk's suggestion that river otter plantar pads may serve a mechanical function. River otter plantar pads are 6–8 mm in diameter, much larger than those of other mustelids, which are 2–4 mm; morphological scaling plays a role, with the river otter weighing about 3–5 times more than the American marten or fisher.^10^ However, Buskirk further observed that, unlike other mustelids, the plantar pads in river otters exhibit disc‐shaped “clusters of fused columns,” of material similar to human cuticle, protruding from the epidermis and that were glabrous (fur‐less) year‐round. In 1987, Melquist and Dronkert hypothesized that river otter plantar pads may provide additional traction on slippery surfaces,^11^ an intriguing idea that we explore in this study.

The river otter paw has additional features that we hypothesize enable it to grip wet surfaces. Surrounding the plantar pad is a soft, grooved epidermal layer. To better understand how the pad may work, let us consider a few examples of other materials used to grip wet substrates. A car's grooved rubber tire is designed to work in both dry and wet environments. Tires prevent hydroplaning, a scenario where a trapped water layer creates a hydrodynamic lift force on the tire and significantly decreases the friction coefficient between the tire and road.^12^ The grooves on the tire allow the underlying fluid to leak out, helping the tire maintain contact with the road. Another example of a gripper is the human fingertip. The grooves of the human fingerprint act as microfluidic channels, inhibiting hydrodynamic lubrication and enabling moisture regulation to maximize friction in wet scenarios.^13^ When soaked, human fingertips wrinkle,^14^ increasing groove width and depth. This adaption, like car tire treads, helps channel away fluid, enhancing surface–substrate contact and increasing friction.

The otter's plantar pad has a similarity to footwear designed specifically for walking on riverbeds. Humans have long waded through rivers for fly‐fishing. Since the mid‐twentieth century, anglers have used felt on the soles of their boots to grip onto wet river rocks. Felt is a textile produced by matting, condensing, and pressing fibers together. Felt boots are now controversial and banned in several US states because of their ability to trap and transport invasive organisms.^15^ Why felt boots work on wet surfaces is poorly understood. We surmise that the felt swells and softens in water. Then, when the person applies weight, the pores in the felt release trapped water, allowing the felt to wrap around asperities in the substrate. Much like felt, the softness of the otter's plantar pad may help to increase the contact area with rough surfaces and push away trapped fluid and biofilms.

In this study, we hypothesize that the river otter plantar pad and surrounding textured tissue improve grip in wet, slippery environments. We begin with describing our methods for studying live otters and characterizing the material properties of the paws of deceased otters. We then report the time of contact of an otter walking on dry land. We use this time‐scale to estimate the possibility of water escaping from the pad during walking on underwater surfaces. We report the histology of the tissue and the results of material testing. We visualize the flow of water from underneath the pads of deceased otters and compare these results to simulations. Lastly, we discuss how the otter paw might give insight into other grip mechanisms and propose areas for future work.

## MATERIALS AND METHODS

### Kinematics

In the summer of 2019, we used a Canon EOS‐1D to film a male North American river otter at Dauset Trails Nature Center in Jackson, Georgia. Fiducial marks were provided by a vertical acrylic plate in the background. Using the Tracker Video Analysis and Modeling Tool software, otter walking kinematics were measured across three trials. The substrate was flat, dry soil in the otter's outdoor enclosure.

### Otter samples

Dauset Trails Nature Center provided two rear paws from deceased North American river otters (age and sex unknown). One paw was a taxidermied adult sample, and the other paw was a frozen juvenile sample.

### Surface roughness and water content

The epidermal layer from the taxidermied otter paw was inspected under a Leica DVM6 microscope with multifocus capability. The multifocus images are shown as 3D images in the LAS X software for the Leica DVM6, which enabled contour mapping and custom line profiles of the surface to determine surface roughness. To measure water content, one plantar pad was excised from the frozen otter paw and was defrosted to room temperature over 10 min. Once defrosted, deionized water was pipetted onto the surface to hydrate the plantar pad. Next, the plantar pad was lifted with tweezers, and excess water was allowed to drip off the sample. The sample was then weighed and placed on an open‐top tray to dry in the lab for 48 h. After 48 h, the dried sample was weighed again, and the water content was calculated.

### Nanoindentation of plantar pad

We used a Hysitron TI 900 triboindenter with 100 µm spherical probe to perform nanoindentation of the excised plantar pad sample of the juvenile otter paw. Peak indentation depth was set at 250 µm. Indentation rate was set at 0.03 µN/nm.

### Fluid drainage experiments and simulation

We used a scalpel to excise a toe pad from the juvenile otter paw sample. The toe pad was then placed in a UV‐fluorescent dye solution bath atop a clear polycarbonate pane. The UV dye was Sigma‐Aldrich fluorescein sodium salt (F6377‐100G). Underneath the pane, we mounted a UV Dino‐Lite USB microscope (equipped with UV LEDs) to visualize the flow while a Mark‐10 ESM‐301 force instrument pushed the sample against the pane.

We modeled steady‐state flow through the grooves of both an otter toepad and a human fingerprint using COMSOL Multiphysics laminar solver. Two‐dimensional maps of the otter paw and human fingerprint were used: specifically, otter paw groove structure was obtained from microscopy and human fingerprint grooves were obtained from Ju and Seo,^16^ a study on digital fingerprint identification. From Ju and Seo, we chose a fingerprint whorl pattern that most resembled the pattern on the otter paw. To simplify the analysis, we set the simulated print profile to a height of 0.5 mm, which is 10 times the natural groove height. This height reduces viscous stresses in the third dimension by a factor of 10, thus making the flows two‐dimensional.

In the simulations, we considered the paw approaching the substrate. The “inlet” for the fluid is the substrate, defined by the plane *z* = 0. It provides a constant fluid velocity, normal to the paw face. Since the height of the grooves is only 5 mm, the incoming fluid changes direction to travel two‐dimensionally along the grooves to exit radially from the perimeter of the paw. Besides the inlet, the groove surfaces were characterized by the no‐slip boundary condition. The tetrahedral mesh was configured to encompass a channel width spanning 8–10 cells, with the entire region modeled using 1 million cells.

To maintain constant flow rate, the conditions of inlet velocity were U=10mm/s (Re = 5) for the otter paw and U=4mm/s (Re = 2) for the fingerprint, with the Reynolds number Re=ULν, where L is the width of the groove and ν is the kinematic viscosity of water. Based on the width of the pad and fingerprint, assumed to be 10 mm, the total flow rate into the grooves was 6 mL/min.

### Histology of plantar pad and surrounding textured epidermis

The 24‐step protocol for the processing of the otter paw was a custom protocol created by A.T.A. of the Georgia Institute of Technology. The frozen juvenile otter paw was placed into a closed‐lid container filled with VWR 10% neutral buffered formalin and fixed for several days in the refrigerator. The volume of the fixative was nine times the volume of the sample. A 5‐mm punch biopsy was removed from the paw ensuring that all layers of the tissue were captured. The tissue was placed into a VWR embedding cassette and stored in 70% VWR reagent alcohol until processing. Processing was completed using an Intelsient RTPH 36 Tissue Processor.

General Data Embedding Center equipment was used for embedding. The sample was embedded in Epredia Histoplast PE paraffin using a medium metal mold. The metal mold was filled with the molten paraffin, the tissue was positioned so cross‐sections would display all layers of the tissue. Coverslips used were VWR 24×55 mm, with Epredia Cytoseal 60. A Microm HM S33 microtome was used for sectioning. Sectioning was performed using a Tissue‐Tek low‐profile blade. The samples were cut at 5 microns thick. A Leica Autostainer XL was used for staining. The reagents used were: VWR xylene, VWR xylene substitute, VWR reagent alcohol (100%, 95%, 70%), Harleco Gills III hematoxylin, VWR Scott's tap water solution, and Epredia Eosin‐Y with phloxine (alcoholic). Total program time without draining was 47 min, with an agitation rate of 10 dips per station. The hematoxylin and eosin (H&E) protocol for staining is shown in Table S1. Note that steps 2–3 and 4–6 were conducted with different stations in order to maintain the purity of the solution.

## RESULTS

### Kinematics

**FIGURE 2:**
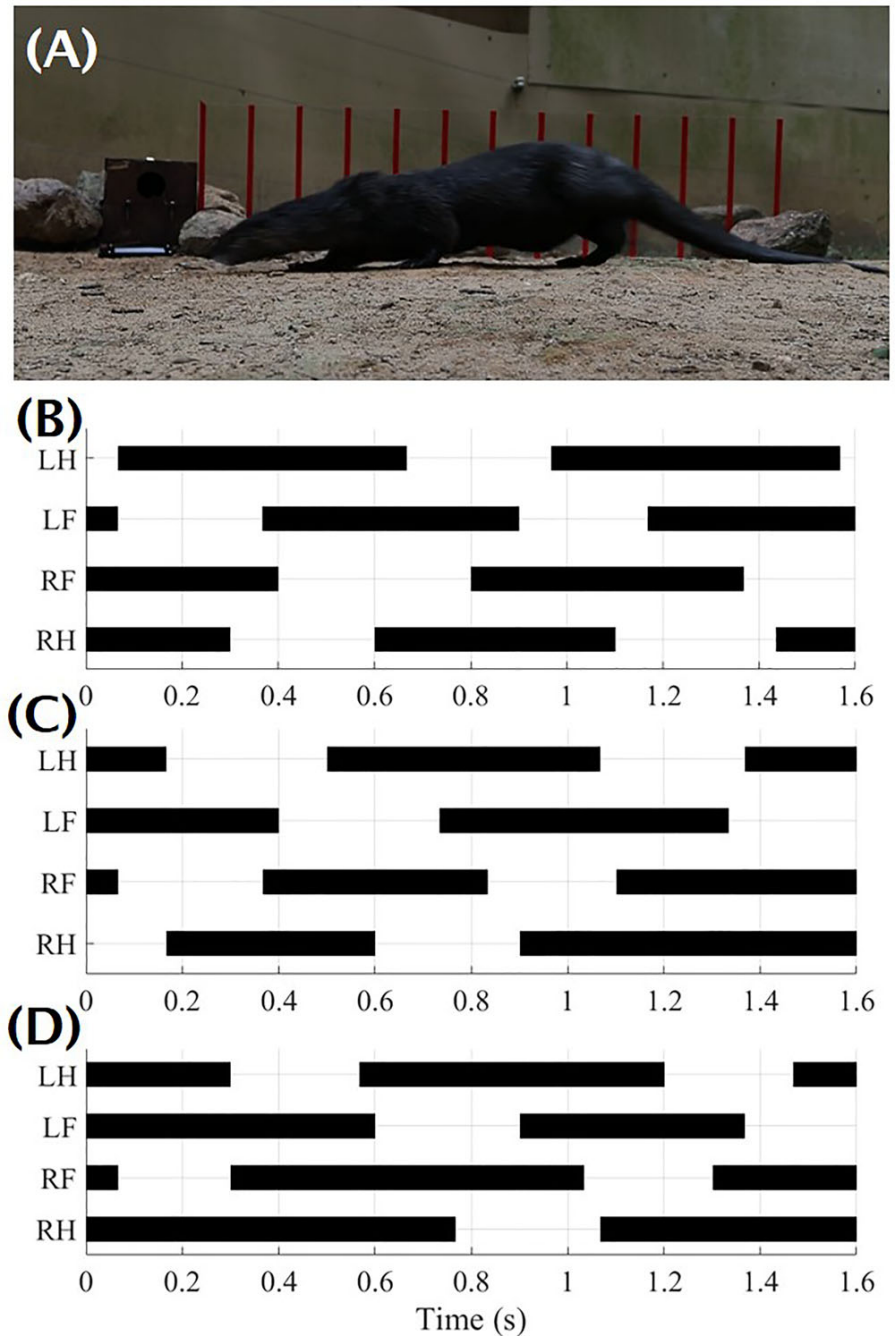
(A) Gait analysis of an otter walking on dry ground. Gaits include (B) steady walking at 0.44 m/s, (C) walking to a complete stop at 0.55 m/s, and (D) complete stop to walking at 0.33 m/s.

To estimate the body weight distribution for each paw, we filmed a male North American river otter (estimated otter body weight at 8 kg) walking on dry ground (Figure 2A). We were unable to film the otter on wet surfaces, so we used the kinematics on dry ground as a proxy. We observed an acceleration from rest, steady‐state walking, and then a deceleration to rest. Figure 2 B–D shows the gait graph, in which the solid line indicates the stance phase, where the foot contacts the ground, and empty spaces indicate the swing phase, where the foot is lifted. The adult otter walks with a symmetric gait at an average speed of 0.45 ± 0.13 m/s, a stride length of 0.41 ± 0.04 m, 4.5 ± 1.7 strides per second, and a paw–ground contact time of 0.3 ± 0.02 s.

In Figure 2C, the otter decelerates at  $0.4\;m/s^{2}$, and in Figure 2D, accelerates at $0.47\;m/s^{2}$. Since the acceleration of the otter is 20 times less than gravity, walking on land likely does not require strong specializations for grip. Specialization for grip may be needed for running, traveling up or downhill, or on wet surfaces.

### Structures of the rear paw pad

We dissected two river otter paws. We report on the white, callous‐like protrusion called the plantar pad and the soft, textured epidermal layer surrounding the plantar pad, as shown in Figure 1A–D. An otter has five webbed toes and four plantar pads. For the adult otter, the plantar pads are 6–8 mm in diameter, and for the juvenile otter, the pads were 5 mm in diameter.

In comparison, a cat's paw has more pads, including four digital pads, a large central metacarpal pad, and a more proximal carpal pad.^17^ A cat's pads comprise most of its contact with the ground, while the otter's plantar pads appear unduly small if they are to be involved in grip. The otter's four plantar pads are 6–8 mm in diameter, comprising a total area of 2 cm^2^, or only 1/7 of the paw. We surmise that growing the pad incurs some tradeoffs due to maintenance and wear of the soft pads.

The otter has four paws, each of area A = 14 cm^2^, which as we will show is large for its body size compared to a cat and human. We calculated the nominal normal stress, which is the normal force divided by the observed area. The true normal stress will be spread across microscopic contacts, which are more difficult to measure. The otter's nominal normal stress per paw while standing (with four paws on the ground) is approximately σ=mg/4A=14.0kPa. During its gait with three paws on the ground, the otter will experience σ=mg/3A=18.6kPa on each paw. In comparison, a human of average weight of 71 kg and foot area of A = 100 cm^2^ would experience σ=mg/2A=34.3kPa while standing. This value is several times that of the otter. A cat of 4.2 kg, which is half the weight of the otter, has paws that have less than seven times the otter's area at A = 1.6 cm^2^. Thus, the cat would experience a normal stress of σ=mg/4A=64.3kPa. The otter's larger paw was likely an adaptation for swimming. Comparing the three normal stresses of an otter, human, and cat, the otter's relatively larger paw area reduces the stress on each paw. Is lower stress beneficial for grip? The short answer is: it's complicated. On the one hand, the otter paw's reduced deformation may be worse for hugging asperities and may lead to a poor grip. On the other hand, the lower stress is associated with a greater area, which increases the chances of finding asperities where it can make Hertzian contact and reduce slip. The relatively larger area may make up for the reduced stress.

We note that this calculation does not consider the plantar pad in contact. If the otter were to put all its weight on its plantar pads with four paws on the ground, the stress would be seven times higher at 98.1 kPa, which is closer to the stress on the cat's paw. More careful work would be needed with live otters to measure the true area of contact and to see the extent that the toes are involved.

We hydrated the juvenile pad and observed that it swells and exhibits fissures as shown in Figure 1E. We speculate that during contact with rough surfaces, these fissures help increase surface area (and, therefore, grip) by having the soft ridges of the plantar pad envelop rough surfaces.

**FIGURE 3:**
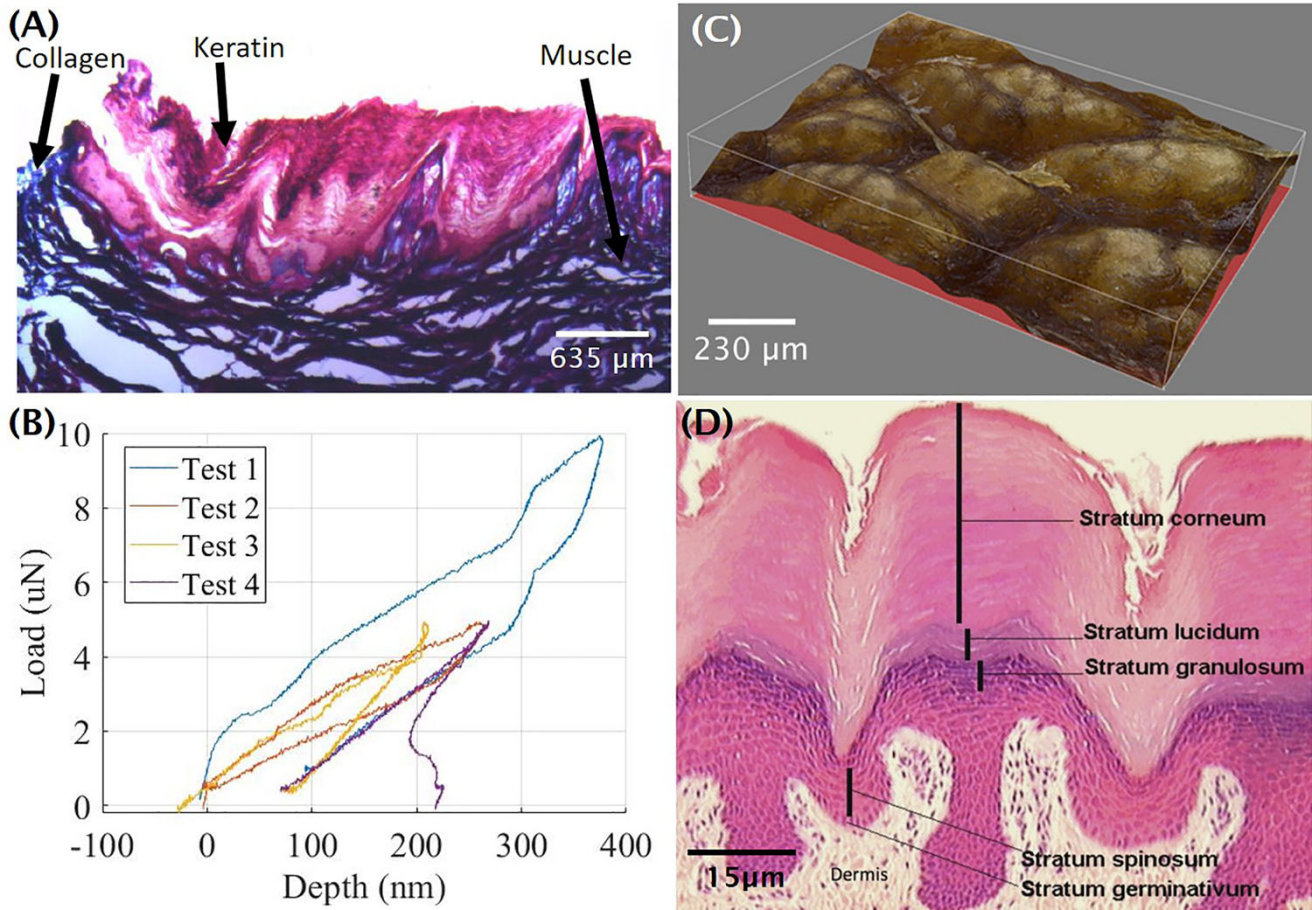
(A) Histology of the plantar pad shows soft keratinized growth. (B) Nanoindentation of the plantar pad at peak indentation depth of 250 µm at 0.03 µN/nm reveals a Young's modulus of 3.075 MPa. (C) 3D map from microscopy of the textured epidermis shows a mean groove depth comparable to those of human fingerprints. (D) Histology of the epidermal layer shows tissue layers analogous in structure to human skin.

We excised several plantar pads from the juvenile otter paw. The hydrated plantar pad was found to be 44.9% water by weight. We performed histology on another part of the otter paw to determine the composition of the plantar pad. The histological section shown in Figure 3A was stained with Masson's trichrome. This stain reveals muscle fibers, keratin (red), and collagen (blue). Based on the staining color observed, we conclude the plantar pad is composed of keratin and water, and the surrounding tissue is composed of muscle and collagen.

We used a nanoindenter on another juvenile otter plantar pad to determine its modulus of elasticity. The sample was hydrated and the nanoindenter spherical probe diameter was 100 µm. The relationship between displacement and force is shown in Figure 3B. The Young's modulus of the tissue indicates its stiffness in the linear regime. The plantar pad has a Young's modulus of 3.075 ± 0.738 MPa, which is more than 10 times softer than the hydrated human stratum corneum (the outermost layer of skin), which has a modulus of 50 MPa.^18^

We excised the epidermal layer surrounding the plantar pad from the juvenile otter paw and performed histology to determine material composition. The histological sections of the epidermal layer shown in Figure 3D were stained in the following manner: H&E stain illustrates nuclei (blue‐purple), erythrocytes (bright pink to red), and cytoplasm (various shades of pink). Masson's trichrome stain shows nuclei (black). The stratum corneum is thick and textured, and is comparable to human skin.

### Flow through grooves on the paw pad

A sample of the epidermal layer was taken from the taxidermied otter paw around the plantar pad and inspected under the Leica DVM6 microscope using the 3D mapping and multifocus capabilities, as shown in Figure 3C. The epidermal layer roughness average (Ra) was 22.7 µm and the RMS roughness (Rq) was 27.3 µm. The toe pad had an Ra of 15.2 µm and an Rq of 18.7 µm. The average groove depth around the plantar pad was approximately 50 µm, which is on par with human fingerprint depths of 59.0 µm.^19^

Studying grip on wet surfaces is a complex subject that involves both visible drainage of fluid as well as clearance of a molecular layer of water, especially for hydrophobic surfaces like textured skin.^20^ These variables can be controlled for by surface chemistry and roughness. We focus the remainder of our study on the drainage of fluid, thus neglecting these important microscopic features that can influence grip.

**FIGURE 4:**
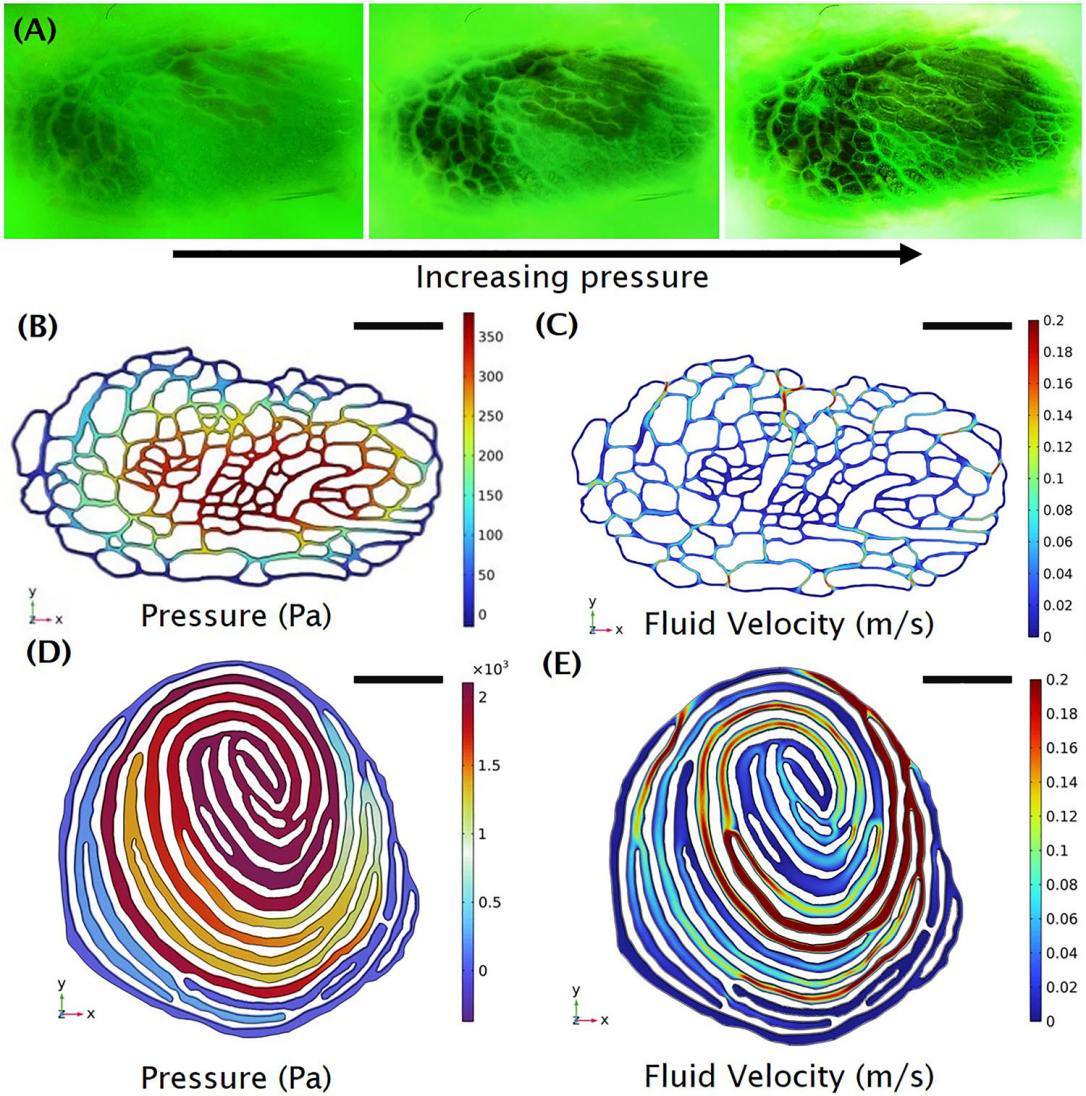
(A) Otter toe pad under 0.9 kgf of compression in UV dye. (B) Simulation of fluid evacuating through an otter toe pad showing pressure and (C) the velocity of the escaping fluid. (D) A human fingerprint under compression showing pressure and (E) fluid velocity.

By compressing a submerged toe pad against a transparent surface, we visualized how the fluid evacuates through the toe pad grooves (Figure 4A). Unlike human fingerprints which have groove patterns mainly in the azimuthal direction (Figure 4D), the groove patterns across the otter toepad are both radial and azimuthal. We hypothesize that otter paw groove patterns better enable the evacuation of fluid, similar in function to tire treads. We test this hypothesis experimentally and in simulation. In Figure 4A, the toe pad is pressed down with increasing force, up to 8.8 N. Initially, the toe pad is submerged and a thin layer of water covers the entire toe pad. As the downward force is increased, this fluid layer is channeled outward along the grooves, shown by the bright rivulets marking the passage of dye.

To better understand the fluid drainage, we turn to a simplified model of flow in a two‐dimensional otter paw, and for comparison, a human fingerprint. The pressure and velocity field through the grooves of the otter toe pad is shown in Figure 4 B,C and for a human fingerprint in Figure 4 D,E.

The fingerprint grooves of 0.2 mm width are nearly four times that of the otter paw, with a groove width of 0.06 mm. Before discussing the flow, we note a few key geometric differences between otter paws and fingerprints. While human fingerprints vary, they generally consist of loops and whorls, which are oriented in the azimuthal direction. They thus differ fundamentally from otter paws, which have grooves in both the azimuthal and radial direction. The white regions in the figure indicate the flat parts of the otter paw (Figure 4 B,C). To grip, the otter should press these flat parts to the substrate and drain fluid through the grooves as quickly as possible. The otter paw is mostly covered in flat parts so it is mostly white. Conversely, the fingerprint has wider grooves and thus fewer flat parts. For the otter, the radial connection between the grooves also implies a topological difference between human fingerprints: specifically, the otter's network of grooves prevents any “dead ends” for the escaping fluid.

For both cases, the center‐most grooves have the highest pressure and lowest flow rate, as to be expected as the central fluid travels the longest distance to escape. Both simulations satisfy the conservation of mass whereby they have a constant flow rate vertically into the page. For the fluid to escape the human fingerprint, it must travel along each groove before traveling radially and entering the next groove. This process continues for several grooves before the fluid escapes. In contrast, the trapped fluid in the otter print can more easily travel radially to escape.

The effectiveness of the otter groove network structure can be seen in the low variability of the velocity fields. Otter grooves have nearly constant velocity (indicated by the large amount of blue), whereas the human fingerprint has variable zones showing “traffic jams” for escaping fluid. A human fingerprint is similar to a highway with no exit ramps, and an otter fingerprint has a large number of exit ramps. We thus conclude that the otter's radial grooves are used as “escape paths” that reduce the resistance to the flow, and enable the paw to make solid contact faster.

Comparing the maximum static pressure, the fingerprint has a higher pressure drop than the otter paw (2100 Pa for the fingerprint and 380 Pa for the otter paw) under the same flow rate. The wider human grooves should reduce the pressure to travel according to Poiseuille's law, which states that pressure scales as $\mathrm{flow}\;\mathrm{rate}/\mathrm{radiu}s^{4}$. However, the tortuosity of the human fingerprint instead generates a 5.5 times higher resistance.

## DISCUSSION

**FIGURE 5:**
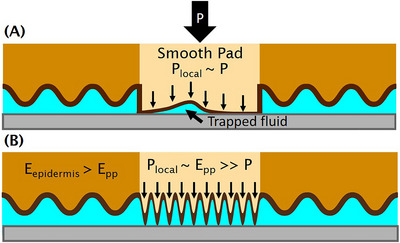
Model of otter paw compression on a substrate. (A) If the plantar pad were smooth, trapped fluid would cause low friction under shear conditions. (B) Fissures on the plantar pad help to evacuate fluid and increase local contact pressures, decreasing time needed to fully evacuate fluid.

We hypothesized that the otter's plantar pad works with the epidermal layer to drain fluid through the grooves, enabling direct paw–substrate contact. The plantar pad protrudes from the paw pad and will likely make contact first, as shown in Figure 5A,B. In this section, we discuss how recent results from mechanics can help make sense of the material properties observed.

### Water evacuation from rough elastic surfaces

We observed that the plantar pad is covered in fissures. Here, we explore the potential purpose those fissures could serve. Let us consider a simplified model of water squeezing between two stiff parallel plates: elastic de‐wetting (or, dry–dry contact) occurs spontaneously when the fluid gap reaches 100 nm. The fluid has to evacuate to reach this small value. To understand this process, we begin by considering the simplest case of a smooth plantar pad compressing onto a smooth substrate, as shown in Figure 5A. Yashima et al.^21^ found that for smooth hydrogels pressing into a plate, fluid can become trapped at the gel–substrate interface. This thin layer of trapped water provides a homogeneous pressure distribution P, leading to low friction coefficients upon shear (i.e., hydrodynamic lubrication, also known as hydroplaning).

How long would it take for a smooth plantar pad to evacuate the trapped fluid when under compression? Consider a Newtonian fluid compressed between parallel plates of diameter D, where pressure P generates flow that is resisted by viscosity μ. Persson^22^ derived the time t to bring the plates together an initial distance h:

(1) $$t=\frac{3\mu }{16P}\;\frac{D^{2}}{h^{2}}$$

Let us apply this result to an otter plantar pad. If the otter applies a constant normal force F against the underwater substrate, the pressure is $P=4F/\pi D^{2}$ and, according to Equation (1), the time is $t∼D^{4}.$ Thus, the time to close the gap decreases very quickly as the plantar pads decrease in size. This may be one reason why the plantar pads are so small.

Let us consider the time it takes for contact between a theoretical smooth plantar pad of D = 8 mm and a wetted smooth and flat surface (no biofouling). From the kinematic analysis, with an otter weight of 8 kg performing a walking gait of three paws on the ground at a time, and four plantar pads on each rear paw, the normal pressure on each plantar pad is P = 18.6 kPa. If we assume a completely smooth plantar pad as shown in Figure 5A, the time it takes for the paw to compress h=1mm of water to 100 nm is t = 57 s. This stepping time is far too long for the otter to walk since our kinematic videos showed the paw contact time is only 0.3 s. Water would become trapped between the smooth pad surface and the substrate, meaning the paw would experience lubrication with the river bed and the otter would certainly slip.

Now, let us consider a fissured plantar pad. While the normal pressure on the bulk plantar pad remains at *P* = 18.6 kPa, the local contact pressure on the peaks of the fissures will be much higher due to the reduced contact area. Similar to Yashima et al.’s^21^ study with textured polydimethylsiloxane (PDMS), the fluid in between the fissure peaks will not carry the load as it evacuates, leading to local contact pressure $P_{local}$ at the fissure peaks scaling with the elastic modulus of the plantar pad $P_{local}∼E_{pp}$, which may be significantly greater than the applied pressure P, as shown in Figure 5B. This scaling assumption only holds if the true contact area is much smaller than the apparent contact area. Additionally, we assume that the textured epidermis surrounding the plantar pad does not compress as much as the plantar pad itself, enabling evacuation of the fluid. In this study, we assume that the Young's modulus of the surrounding epidermis is greater than the plantar pad $E_{epidermis}>E_{pp}$.

Using Equation (1), how long would it take for the fissured plantar pad to evacuate fluid and contact the substrate? While we do not know the actual contact area of the fissured plantar pad to the substrate, or the width of the fissures themselves, we can make some assumptions. Based on the local contact pressure logic, the fluid pressure P is $P_{local}∼E_{pp}=3.075\;\mathrm{MPa}$. Substituting this higher‐pressure $P_{local}$ into Equation (1), the time to evacuate the fluid drops to t = 0.35 s, which is on par with the foot contact time of 0.3 s when the otter walks on land. If we were to add fissures (or areas for fluid to escape), this time would drop significantly more since the total plate contact area would be smaller. In reality, the apexes that contact the substrate would be significantly smaller than the diameter of the whole plantar pad, decreasing contact time further. While the textured plantar pad helps with contact and fluid evacuation, realistically the substrate will be biofouled. Biofouled substrates that have a high viscosity μ will increase t, making it more difficult for the otter to make solid–solid contact. The otter will need to walk slower to maintain grip with slippery river rocks.

### Scaling

We have shown that the otter paw is heterogeneous, with soft plantar pads interposed in a wider textured epidermis of greater stiffness. In this section, we consider that a soft pad might better envelope asperities. By enveloping an asperity, the paw can use normal forces to resist sliding. A similar principle is likely behind athletic footwear and tires that prevent slip. However, the white pads appear softer than the soles of normal athletic shoes. Why? Let us consider the scaling of pad deformation with body size.

Consider a pad with area A and Young's modulus $E_{pp}$. The pad will experience a deformation δ if given a compressive force $F=\sigma A=E_{pp}\;A\delta /h,$ where h is the thickness of the pad. By translating this relation to Hooke's law, F=kδ, the effective stiffness of this pad is $k=E_{pp}\;A/h$. If the animal's weight is mg, then by Hooke's law, the pad will deflect an amount $\delta =\mathrm{mgh}/E_{pp}A$. Basically, an animal's weight provides the force to compress the pad, but the area of the foot dictates the pressure on the pad.

For simplicity, we first consider isometry where body mass scales as $m∼L^{3}$ and the pad area scales as $A∼L^{2}$, and h∼L where L is the characteristic body length. The deflection then scales with the square of body size: $\delta ∼L^{2}g/E_{pp}$. Assume the environmental asperities remain of fixed size for all animals that walk on it. Thus, if smaller animals want to envelop an asperity of fixed size δ, they should have a lower stiffness E to compensate for their lower body size L. Specifically, $E_{pp}∼L^{2}g/\delta ∼L^{2}$. Thus, across animals, $E_{pp}/L^{2}$ should be constant. Furthermore, otters are walking underwater where they have some partial weight support by buoyancy. Given that their effective weight is lower, they must have an even lower stiffness E for their paw pads than terrestrial animals.

Let us see if this is the case. An otter's weight is 8 kg, a human's is 71 kg, and the ratio of length scales is roughly 3. If $E_{pp}/L^{2}$ is constant, then $\frac{E_{otter}}{{L_{otter}}^{2}}=\frac{E_{human}}{{L_{human}}^{2}}$ and $\frac{E_{otter}}{E_{human}}=\frac{{L_{otter}}^{2}}{{L_{human}}^{2}}$ = 1/9. Thus, we expect the human heel pad to be nine times stiffer than the otter's. The human heel pad is filled with fatty tissue, giving it a Young's modulus of 265 kPA = 0.3 MPa, which is actually 10 times softer than the otter plantar pad.^23^ This is in direct violation of the scaling law that we proposed.

We can try another argument by considering the nominal area *A* and true area $A_{local}$ beneath the fringes in the plantar pad. The force *F* supported by the foot is actually supported by the local area of the fringes: $F=P_{local}\;A_{local}=AP$. The argument from Yashima et al.^21^ for soft textured contact states is $P_{local}∼E_{pp}$. If we assume that we have close to 100% contact area for compliant contact, $A_{local}∼A∼L^{2}$. If the body weight is again supported $F∼Mg∼L^{3}$, we find a similar result (albeit a different exponent) $E_{pp}∼L$, which still suggests that otter paws should be stiffer than human pads, not softer.

The fluid mechanics of flow evacuation, rather than the enveloping of asperities, may be the primary reason for the otter paw pad material properties. Also, our method of stiffness measurement was done at a small length scale and may not be comparable to the experiment on the human plantar pad. Additionally, we recognize there may be tradeoffs: tissue softness helps envelop local asperities, while stiffness helps with fluid evacuation.

Furthermore, otter paw sizes do not fall alongside predictions for mammals. Measurements of carnivores have found that paws are not isometric, but that smaller animals have larger paws for their size. Moreover, larger animals tend to have stiffer paws. In fact, the scaling trends of Chi and Louise Roth^24^ suggest that the otter of mass 8 kg should have a total paw area of 1700 mm^2^, which is three times smaller than our measured total paw area of 5600 mm^2^. We suspect the otter's unexpectedly large paws may have to do with its swimming ability.

## CONCLUSION

In this study, we studied the unique grooved soft structures on the otter paw. We saw that the radial grooves of the otter toe pad evacuate fluid better than human fingerprints, which only have azimuthal grooves. We applied fluid mechanics models to show that the plantar pad's softness can help fluid evacuate and to maintain grip. By studying otters, we might inspire better ways to grip slippery materials. Antislip mechanisms could be useful in marine environments, in the kitchen, in sports, and in medicine. Felt‐soled boots have been banned in many states due to the spread of invasive species; by learning how otters grip onto river rocks, we can learn how to design a better river boot.

## AUTHOR CONTRIBUTIONS

This study was led by A.N., who coordinated the experiments and wrote the paper. J.L., B.S., and M.T. conducted the experiments. A.T.A. conducted the histology. J.N. supplied the laboratory space and equipment for specimen analysis and reviewed/edited the paper. S.K. conducted the numerics for this study. D.L.H. contributed to the scaling principles and writing the paper.

## COMPETING INTERESTS

There are no conflicts of interest.

### PEER REVIEW

The peer review history for this article is available at: https://publons.com/publon/10.1111/nyas.15263

## Supporting information



Table S1: H&E protocol for histological staining of otter paw sample.
