# Supplementary material for: Enhanced wet grip with North American river otter paws
Source: Ann N Y Acad Sci. 2024 Dec 12;1542(1):638–46. doi: 10.1111/nyas.15263 (PMC11668502; doi:10.1111/nyas.15263)
Supplement: Supplementary file 1 — Table S1: H&E protocol for histological staining of otter paw sample. [file NYAS-1542-638-s001.docx]

Table S1: H&E Protocol for Histological Staining of Otter Paw Sample

| **STEP** | **STATION** | **REAGENT** | **TIME(min:sec)** | **EXACT** |
| --- | --- | --- | --- | --- |
| 1 | 1 | Xylene | 5:00 | N |
| 2 | 2 | Xylene Substitute | 5:00 | N |
| 3 | 3 | Xylene Substitute | 5:00 | N |
| 4 | 4 | 100% alcohol | 3:00 | N |
| 5 | 5 | 100% alcohol | 2:00 | N |
| 6 | 6 | 100% alcohol | 2:00 | N |
| 7 | 7 | 95% alcohol | 2:00 | N |
| 8 | wash 1 | 70% alcohol | 2:00 | N |
| 9 | wash 2 | Water | 2:00 | N |
| 10 | 8 | Hematoxylin | :45 | Y |
| 11 | wash 5 | Water | 1:00 | N |
| 12 | 9 | Acid alcohol | :01 | Y |
| 13 | wash 4 | Water | 1:00 | N |
| 14 | 10 | Scott's | :30 | N |
| 15 | wash 3 | Water | 2:00 | N |
| 16 | 11 | 95% alcohol | 1:00 | N |
| 17 | 12 | Eosin, alcoholic | 3:00 | N |
| 18 | 13 | 95% alcohol | :30 | N |
| 19 | 14 | 100% alcohol | 1:00 | N |
| 20 | 15 | 100% alcohol | 2:00 | N |
| 21 | 16 | 100% alcohol | 2:00 | N |
| 22 | 17 | Xylene Substitute | 2:00 | N |
| 23 | 18 | Xylene Substitute | 2:00 | N |
| 24 | Exit | Xylene | 1:00 | N |
